# Stability of blocked replication forks *in vivo*

**DOI:** 10.1093/nar/gkv1079

**Published:** 2015-10-20

**Authors:** Karla A. Mettrick, Ian Grainge

**Affiliations:** School of Environmental and Life Sciences, University of Newcastle, Callaghan, NSW 2308, Australia

## Abstract

Replication of chromosomal DNA must be carried out to completion in order for a cell to proliferate. However, replication forks can stall during this process for a variety of reasons, including nucleoprotein ‘roadblocks’ and DNA lesions. In these circumstances the replisome copying the DNA may disengage from the chromosome to allow various repair processes to restore DNA integrity and enable replication to continue. Here, we report the *in vivo* stability of the replication fork when it encounters a nucleoprotein blockage in *Escherichia coli*. Using a site-specific and reversible protein block system in conjunction with the temperature sensitive DnaC helicase loader and DnaB replicative helicase, we monitored the disappearance of the Y-shaped DNA replication fork structures using neutral-neutral 2D agarose gels. We show the replication fork collapses within 5 min of encountering the roadblock. Therefore, the stalled replication fork does not pause at a block in a stable confirmation for an extended period of time as previously postulated.

## INTRODUCTION

Cell viability requires the complete and precise duplication of the entire genome in a timely manner. Replication of chromosomes can be impeded during cell growth by the presence of DNA lesions, excessive or tightly bound proteins on the DNA or unusual DNA structures that obstruct the progression of the replisome (1). If a replication fork encounters any of these roadblocks, the replisome may disengage, at least partially, from the DNA, allowing processing of the DNA into a structure that facilitates reloading of the replication proteins and restart of replication. This process may allow access of DNA repair factors, accessory helicases and homologous recombination proteins which can repair or bypass the blocking lesions. In bacteria, the regularity of the replication fork encountering these impediments that lead to dissociation can be inferred from the key role that the PriA protein plays in the survival of the cell (2), with the most frequent cause of dissociation thought to be nucleoprotein blocks (3). The fate of the replication proteins when encountering such impediments is uncertain, however the replisome is thought to remain stable for an extended period of time at protein roadblocks before it is removed from the DNA (4–6). Similarly, evidence suggests replisomes that have stalled owing to head-on collisions with transcription complexes remain stable for 60 min or more (7).

Initiation of replication of the *Escherichia coli* chromosome occurs at a unique origin of replication, *oriC* (5). The initiator protein DnaA melts an AT-rich region within *oriC* allowing binding of a DnaB-DnaC complex onto each of the separated DNA strands (8). DnaC is essential for the loading of the replicative helicase DnaB onto the DNA but subsequently dissociates when the primase DnaG interacts with DnaB (9,10). The hexameric DnaB encircles the DNA and separates the strands to allow synthesis of the first RNA primer resulting in the assembling of the DNA polymerase III holoenzyme (PolIII). The core polymerases within the holoenzyme are tethered to the separated DNA strands by the β-sliding clamp, a processivity factor, and synthesise the DNA in either a continuous (leading strand) or discontinuous (lagging strand) manner. Subsequently, the circular chromosome is replicated by the two independent replisomes moving bidirectionally from *oriC* (11). While the lagging strand polymerase in each replisome dissociates from DNA upon completion of an Okazaki fragment (12), overall the complex remains bound to the DNA because of the long half-life of the β-sliding clamp (13) and the multiple interactions between polymerases, the clamp loader complex and the DnaB helicase. However, the evidence for the fate of the typically stable replisome upon meeting a roadblock is conflicting. Previously, it has been shown using an *in vitro* nucleoprotein roadblock formed from multiple copies of the *lacI/lacO* repressor/operator that a paused replisome has a half-life of 6 min (14). This is in line with earlier *in vitro* data that a stalled replisome blocked by torsional strain in the DNA has a half-life of 4 min (15). Conversely, *in vivo* data has suggested that a stalled replisome may be stable for hours, suggesting that *in vivo* external factors colocalise with a stalled replisome to prevent this rapid dissociation (4,5). Using a transcriptional repressor protein bound to an array of operator sites in the *E. coli* chromosome, it was seen that replication forks could be efficiently blocked throughout a cell population. When the DNA was examined it was found that Y-shaped DNA was abundant at the array representing a site-specific replication fork block, and the level of the Y-shaped signal remained constant over hours. Furthermore, it was found that within 5 min of addition of the gratuitous inducer for the repressor protein, the replication forks had restarted and replication had moved through the array. It was, therefore, proposed that the replisome remained bound and stable over this period, allowing for the rapid restart of replication.

Here, we have investigated the *in vivo* stability of the replisome at a site-specific protein roadblock created in *E. coli*. A temperature sensitive allele of the *dnaC* gene (*dnaC*2) was used to prevent reloading of the replisome once dissociation occurred. A temperature sensitive allele of the DnaB replicative helicase was also used to rapidly inactivate the replisome. The timing of DNA replication fork collapse and subsequent processing of the DNA in these mutants and in a wild-type strain was visualised by neutral-neutral 2D agarose gels. Our results show that the replication fork collapses rapidly upon encountering the roadblock with a half-life of <5 min, suggesting the arrested replisome at a nucleoprotein roadblock *in vivo* is more transient than previously supposed, and is more similar to the *in vitro* situation.

## MATERIALS AND METHODS

### Bacterial strains and plasmids

Bacterial strains used in this study were derivatives of *E. coli* K12 AB1157 (16) carrying an array of 240 copies of *tetO* (17). Temperature-sensitive alleles were introduced by P1 transduction, either *dnaCts* (*dnaC2*) (18) or *dnaBts* (*dnaB8*) (19).

Cells were transformed with a plasmid (pKM1) which encodes the TetR-YFP repressor under control of the P*ara* promoter. To produce pKM1, the *psi* site from pSC101 was amplified by PCR and inserted into the HindIII restriction site of the previously published pLau53 (17).

### Growth of bacteria

Overnight cultures grown at 30°C in L-broth were diluted to OD_600 nm_ = 0.01 in a dilute complex medium (0.1% tryptone, 0.05% yeast extract, 0.1% NaCl, 0.17 M KH_2_PO_4_, 0.72 M K_2_HPO_4_). Antibiotics were added as required at the following concentrations: ampicillin 100 μg ml^−1^, kanamycin 50 μg ml^−1^; gentamicin 10 μg ml^−1^; tetracycline 10 μg ml^−1^.

Production of the fluorescent repressor TetR-YFP was induced by addition of 0.1% arabinose when cells reached above OD_600 nm_ = 0.05. Cells were then incubated for 1 h and examined using a fluorescence microscope to confirm the extent of replication blockage throughout the population. At least 100 cells of each strain were examined and foci enumerated. Cells were then shifted to 42°C to induce replisome collapse in the temperature sensitive strain *dnaBts* and prevent new rounds of replication in the temperature sensitive strain *dnaCts*. The gratuitous inducer anhydrotetracycline (AT; 100 ng ml^−1^) was used to relieve tight repressor binding. To determine viability, a ten-fold serial dilution was generated and 5 μl of each dilution spotted onto agar containing appropriate antibiotics and anhydrotetracyline if required. The same dilutions were spread to determine CFU ml^−1^. All plates were grown at 30°C overnight.

### Microscopy

For microscopy, cells were transferred to a slide mounted with 1% (w/v) agarose layer and visualised with a 100× NA 1.4 objective on a Zeiss Axioskop2 equipped with a Hamamatsu Orca-AG CCD camera. eYFP was observed through Chroma filter set 41028. The images were taken, analysed and processed by MetaMorph^®^ (Molecular Devices^®^) and Adobe^®^ Photoshop^®^ CS6.

### 2D DNA gels and Southern hybridisation

Samples of cells were taken at the indicated time points, 0.1% (final) sodium azide was added and cells were put on ice. Cells were harvested, embedded in 0.4% agarose plugs and subsequently incubated in EC lysis solution (10 mM Tris–HCl [pH 8], 1 M NaCl, 100 mM EDTA, 0.2% sodium deoxycholate, 0.5% Sarkosyl, 100 μg ml^−1^ lysozyme, 50 μg ml^−1^ RNase A) at 37°C for 2 h. The EC lysis solution was replaced with ESP (0.5 M EDTA, 1% sarcosyl, 1 mg ml^−1^ of proteinase K) and incubation was continued overnight. Following extensive washing, DNA was digested with either EcoRV for visualisation of the array region, or EcoRI for visualisation of the 4.6 kb region directly upstream of the array. 2D gel conditions were as described previously (20). DNA was subsequently transferred to Zeta-Probe nylon membranes (Bio-Rad) and detected using either radiolabelled *tetO* array or a PCR product amplifying the region immediately upstream of the array as probe. Blots of at least two independent experiments were analysed by phosphor imaging with a Typhoon TRIO Variable Mode Imager (Amersham Science) and Adobe^®^ Photoshop^®^ CS6. Replication intermediate DNA was quantified by area and intensity using MetaMorph^®^ (Molecular Devices^®^).

## RESULTS

### A protein roadblock causes replication forks to collapse

To assess the stability of the replisome on the DNA when it encounters an obstruction to replication, a system to create a protein roadblock *in vivo* was utilised. In a strain carrying 240 copies of the *tetO* sequence 15 kb counterclockwise of *oriC*, the arabinose-induced overproduction of TetR-YFP generates a site-specific obstruction that the replisome cannot proceed through (4). The replication blockage was confirmed using 2-D gels that demonstrated a Y-shaped DNA structure resulting from replication being blocked within the first 500 bases of the array. This signal was observed to be stable over 4 h (4). Upon addition of anhydrotetracycline the replication fork blockage was released within 5 min, allowing all the blocked forks to resume replication. Based on this evidence it was previously proposed that the replisome was intact and stable over the 4 h time period.

A temperature sensitive allele of the replicative helicase (*dnaBts*) was introduced into the strain carrying the operator array. Previous studies have shown that at the non-permissive temperature this allele leads to replisome collapse and subsequent fork reversal and processing (21). A strain was also made by addition of a *dnaCts* allele. This allele was initially identified as a ‘slow-stop’ mutant that is able to continue replicating at a non-permissive temperature until the DnaB is required to be reloaded onto the DNA (19,22). In fact, it has been shown that strains carrying *dnaCts* alleles that were initially characterised as ‘fast-stop’ are actually able to continue replicating at non-permissive temperatures, and behave similarly to the ‘slow-stop’ mutants (23,24). These studies indicate DnaC is not necessary for an active replisome, and evidence suggests DnaC dissociates from the DNA once DnaB interacts with DnaG (10); indeed active priming complexes do not contain DnaC (25) and replication has been found to be able to proceed *in vitro* in the absence of DnaC (7,26,27). Therefore, in the *dnaCts* strain used here the replisome is able to continue ongoing replication at non-permissive temperatures if it is not otherwise impeded. However, under these conditions DnaB cannot be re-loaded once it dissociates from the DNA (22).

**Figure 1.**
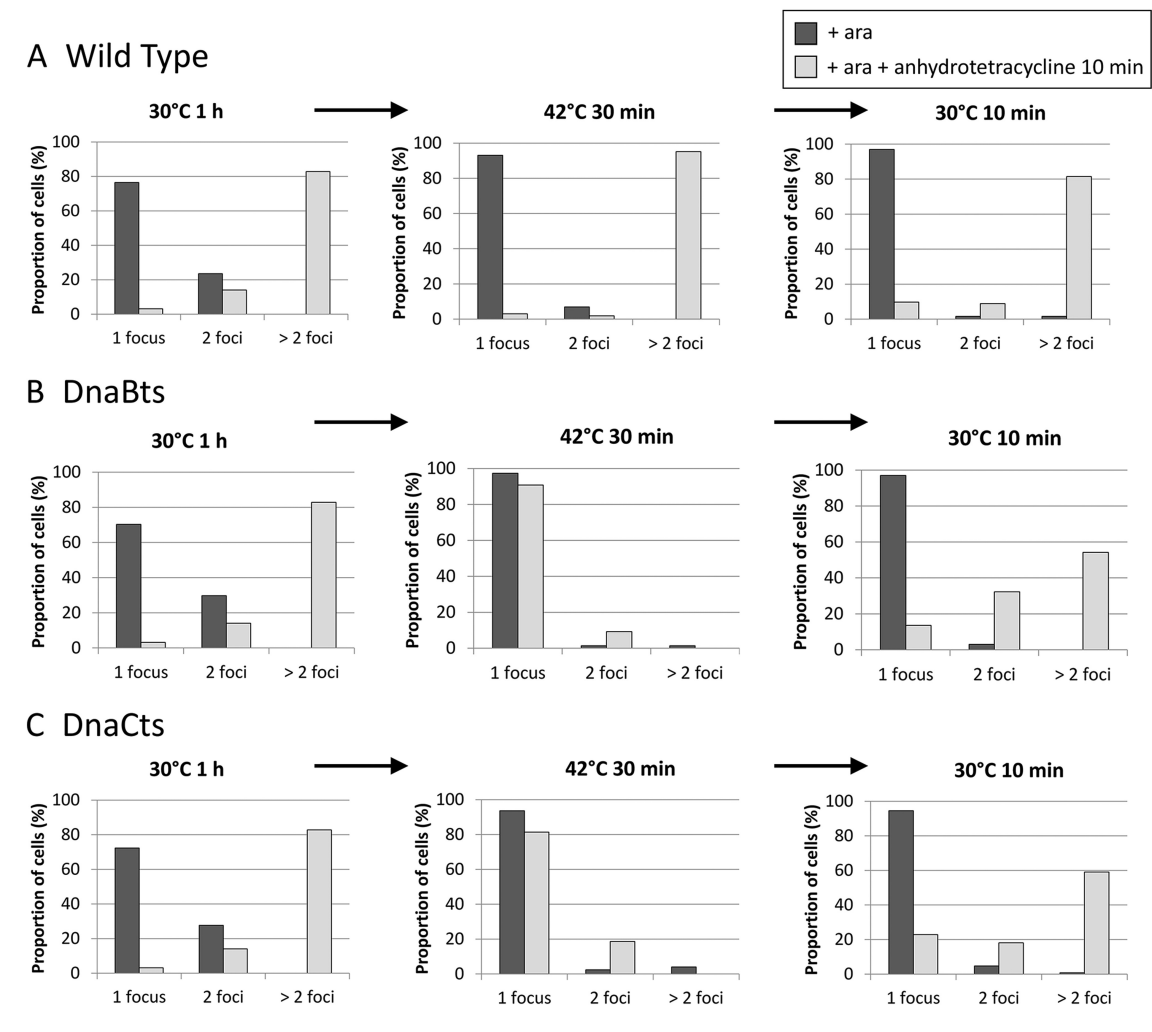
Proportions of cells containing single or multiple foci, representing the *tetO* array, following overproduction of TetR-YFP. Left-most set of graphs: (**A**) wild type, (B) DnaBts or (C) DnaCts cells were grown at 30°C in the presence of 0.1% arabinose (ara) for 1 h (dark grey bars) and then anhydrotetracycline was added for 10 min to a subpopulation to release the replication block (light grey bars). Middle set of graphs: cells that had been treated only with arabinose at 30°C were shifted to 42°C (a non-permissive temperature for DnaBts and DnaC(ts)) for 30 min (dark grey bars) and then anhydrotetracycline was added for 10 min to a subpopulation (light grey bars). Right-most set of graphs: the arabinose-only treated cells (42°C, 30 min) were shifted back to permissive temperature (dark grey bars) and anhydrotetracycline was added for 10 min to a subpopulation (light grey bars). See supplementary material for representative micrographs.

The three strains carrying the replication blocking array were grown and TetR-YFP production was induced for an hour with arabinose. Upon microscopic examination, an average of 73% of the population was deemed to have replication blocked by the presence of one focus per cell (Figure 1). The focus is formed by TetR-YFP binding to the tandem *tetO* sequences within the array. When replication is able to proceed, multiple copies of the array will exist within the cell and multiple foci will be visualised. The proportion of the population with one focus is a comparable fraction to what has been seen previously (5). The remaining population had two foci per cell that were well segregated and the cells were elongated. This suggests the array was already replicated upon induction with arabinose and the round of replication would have completed but the cells have yet to divide. If so, then these cells would not be able to replicate in the next round (see Supplementary Figure S1 for representative images). At this stage the population were deemed to have replication sufficiently blocked to continue the analysis of the effects of the block on foci count and viability. To test whether the replication block could be reversed with the addition of anhydrotetracycline, a sample was taken 10 min after the gratuitous inducer was added and the number of cells having one focus, two foci, or more than two foci were counted. For the replisome to proceed, sufficient repressor has to have been removed from the DNA. Multiple foci within a cell signify the array has been successfully replicated and sufficient time has passed to allow the loci to move apart overcoming any sister chromosome cohesion that was present. The majority of cells in all three strains at 30°C in the presence of anhydrotetracycline were shown to have successfully restarted replication: >80% of cells in each of the strains showed two foci or more.

The cells that had been deemed sufficiently blocked (+ ara only) were shifted to 42°C. After 30 min, the foci number within the cells was determined. The size of the population with one focus was increased in all three strains in comparison to the 30°C sample, confirming the cells with two foci in the former population had indeed been unable to replicate in the next round. The addition of anhydrotetracycline to the cells after they had been at 42°C for 30 min (Figure 1) or 1 h (Supplementary Figure S2) only enabled the restart of replication in the wild-type strain suggesting the replisome was no longer functional in either the *dnaBts* or *dnaCts* strains. Despite the inability of these strains to restart replication at 42°C, when the blocked cells were shifted back to permissive temperature and anhydrotetracycline added, replication was able to restart within 10 min in all three strains (Figure 1). There was a slightly increased percentage of the population with single foci and a slight reduction in the number of cells with >2 foci in the temperature sensitive strains that had undergone a temperature shift in comparison to the corresponding sample that had only been grown at 30°C. This indicates that either replication restart was not able to occur as rapidly after the temperature shift, or possibly at all, in some of the *ts* mutant cells.

**Figure 2.**
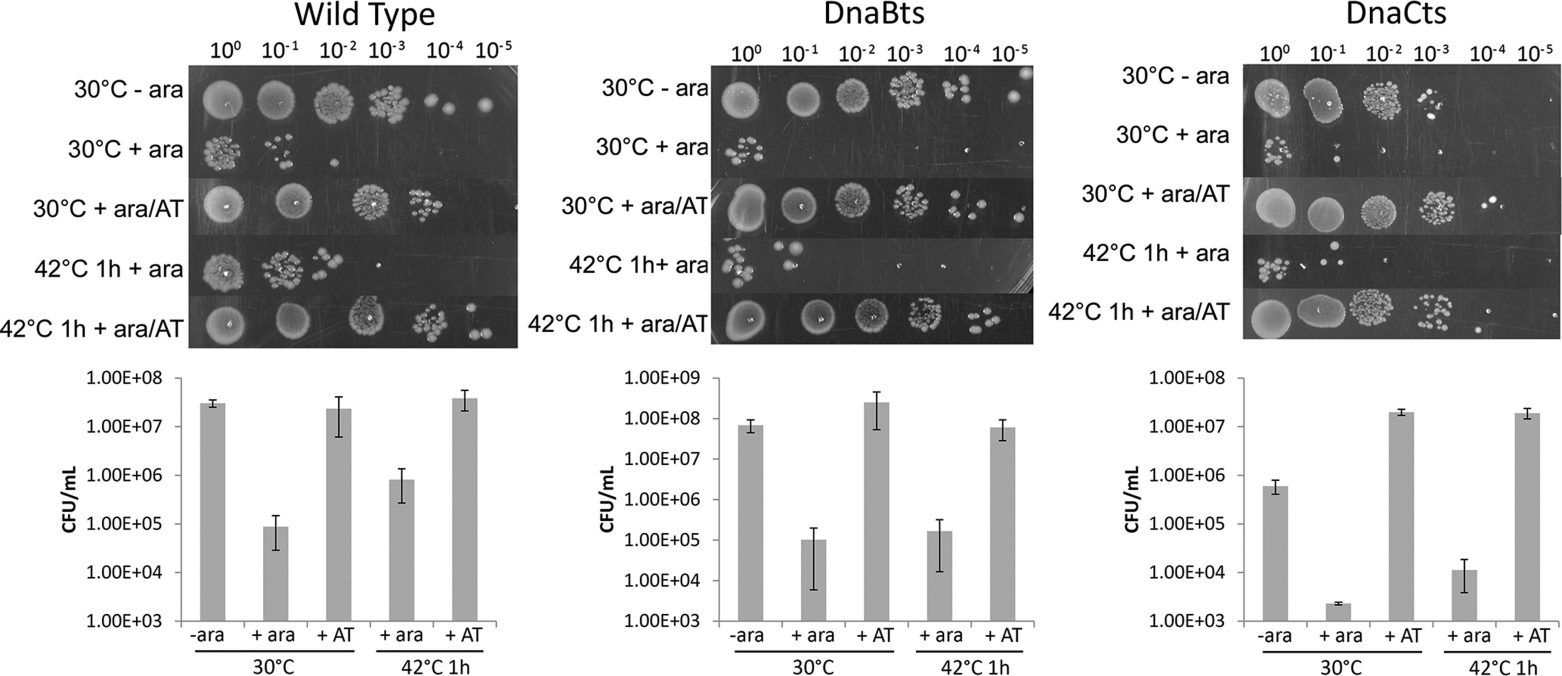
Viability following creation of a replication roadblock. Wild type, DnaBts or DnaCts cells were grown at 30°C in the absence or presence of 0.1% arabinose (1 h) to induce replication blockage. Subpopulations of the blocked (+ ara) cells were either incubated for 10 min in the presence of anhydrotetracycline (AT) or shifted to 42°C (a non-permissive temperature for DnaBts and DnaC(ts)) for 1 h before also being incubated with anhydrotetracycline for 10 min. Cells were serial diluted 10-fold and either spotted or spread onto agar containing ampicillin only (−/+ ara samples) or ampicillin with anhydrotetracycline (+ AT samples) and grown at 30°C to determine cell viability. Top: representative plates showing colonies at indicated dilutions. Bottom: graphs showing the average results +/− SEM.

The effect of replication blockage and restart on cell viability in these populations was also determined. Cells that had been blocked and released at 30°C as well as those subjected to 42°C prior to release of the replication block were spread onto arabinose-free agar and the colonies counted after being incubated overnight at 30°C (Figure 2). Cells with arabinose added (+ara) had considerably decreased viability (2 to 3 orders of magnitude lower) compared to cells that either had never had arabinose added or those that had subsequently been treated with anhydrotetracycline (+AT), due to the replication blockage present. The cells to which anhydrotetracycline was added showed recovery of viability that was nearly equivalent to the non-treated sample (compare -ara to +ara/AT) for all three strains (Figure 2). Cells that had undergone a temperature shift and had the block subsequently relieved did not have viability significantly different to the cells that had not been temperature shifted suggesting that despite a larger population of cells still having 1 focus after 10 min (Figure 1), these cells were still able to restart replication, and no loss of the number of colony forming units occurred.

These results confirm that the reversible replication roadblock was fully functional in all three strains, and that the replisome could be inactivated by temperature shift to 42°C in the *ts* strains. However, once these cells were returned to permissive temperature a full recovery of viability was observed, with the majority of cells showing replicated and segregated foci within 10 min of return to 30°C in the presence of anhydrotetracycline.

**Figure 3.**
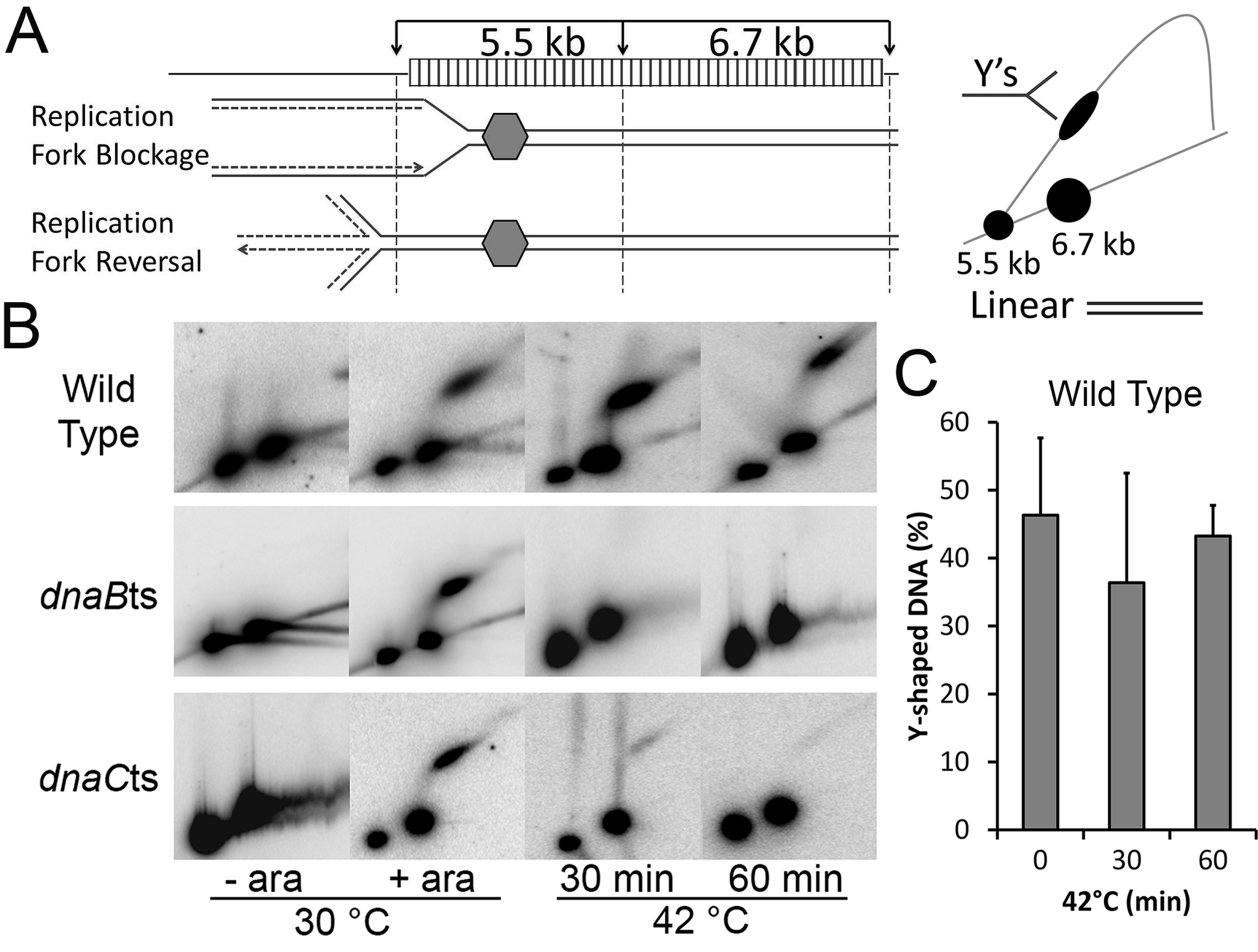
Visualisation of DNA replication fork collapse. (**A**) Schematics of the EcoRV digest of the array region and subsequent signals visualised by Southern hybridisation and a radioactive probe to the array. Replication forks entering the array from the origin become blocked within the 5.5 kb fragment. Cells with a replication block at this position will have the signal corresponding to the 5.5 kb fragment located on the Y-arc. Restriction sites are indicated with arrows. (**B**) 2D gel analysis of EcoRV digested DNA following replication block (+ ara) and subsequent shift to 42°C, a non-permissive temperature for DnaBts and DnaC(ts). (**C**) Percentage of 5.5 kb DNA within the blot located in the Y-arc of the wild type strain (error bars are SEM from three independent experiments).

The structure of the DNA at the roadblock within these cell populations was subsequently visualised using 2D neutral–neutral gel electrophoresis and Southern hybridisation. Digestion of the DNA with EcoRV yields a 5.5 kb and a 6.7 kb fragment of the array region (Figure 3A). At 30°C, the absence of a roadblock means that replication passes through the region unimpeded, and the DNA is almost exclusively seen as linear, visualised as a distinct spot for each of the fragments (Figure 3B). The lower spot represents the 5.5 kb section of the array that is closest to the origin. The presence of arabinose results in the population of the cells becoming blocked at a similar position within the array (4). This is visualised as an elongated spot on the Y-arc. The 6.5 kb fragment remains constant as a spot corresponding to linear DNA as the replication fork cannot progress into this fragment, whereas the intensity of the 5.5 kb spot decreases concomitantly with the increase in Y signal. Replication forks in the wild type strain that had been transferred to growth at 42°C for 30 or 60 min remain blocked at approximately the same proportion at both time points, as shown by the remaining signal on the Y-arc. The DNA signals for the blocked Y and the linear spots were quantified, and the proportion of the signal contained in the Y-shaped structure was calculated (Figure 3C). There is no significant difference between the proportion of Y-signals at the different time points. Therefore, in this strain the temperature shift to 42°C did not appear to affect replication fork stability.

When the same analysis was carried out for a strain carrying *dnaBts*, the prolonged blocked structure was not seen at 42°C (Figure 3B). The elongated spot of Y-shaped DNA indicative of a replication fork blockage is present prominently at 30°C (∼60% of the DNA is in the Y-shaped signal), but disappears within 30 min of the shift to 42°C. This suggests that the forked DNA structure is being processed in some way that leads to the Y-signal being converted back to a linear signal. One possible processing event that could be occurring is replication fork reversal (RFR) which migrates the branch point out of the restriction fragment being examined leaving only linear DNA upon restriction enzyme digestion (Figure 3A). Presumably DnaBts dissociates from the DNA upon shift to non-permissive temperature and the other replisome components may also disengage as a consequence. This leaves the Y-shaped DNA open to processing by other enzymes leading to loss of that signal (RFR or nuclease digestion). The absence of the Y-arc signal at 30 min indicates this happens in all blocked cells within the population within that timeframe.

DnaC does not associate with the replicating replisome (28) and, therefore, its deactivation at 42°C in the *dnaCts* strain should not cause replisome dissociation. However, replication forks that do collapse in this strain should not be able to re-load the DnaB helicase at the non-permissive temperature. Furthermore, new rounds of replication from *oriC* should not be able to able to initiate due to the lack of functional DnaC. Consequently, this variant gives an indication of the stability of replisomes that run into the block in an otherwise wild type strain. It has previously been assumed that the stalled replisome remains associated with the DNA in this type of impediment over the course of several hours (4). Although the *dnaCts* strain produced a level of replication blockage equivalent to the wild type at 30°C (∼68% Y-shaped DNA) (Figure 3B), the Y-shaped structures were seen to disappear at the non-permissive temperature, within 30 min.

### Replication fork collapse leads to replication fork reversal

**Figure 4.**
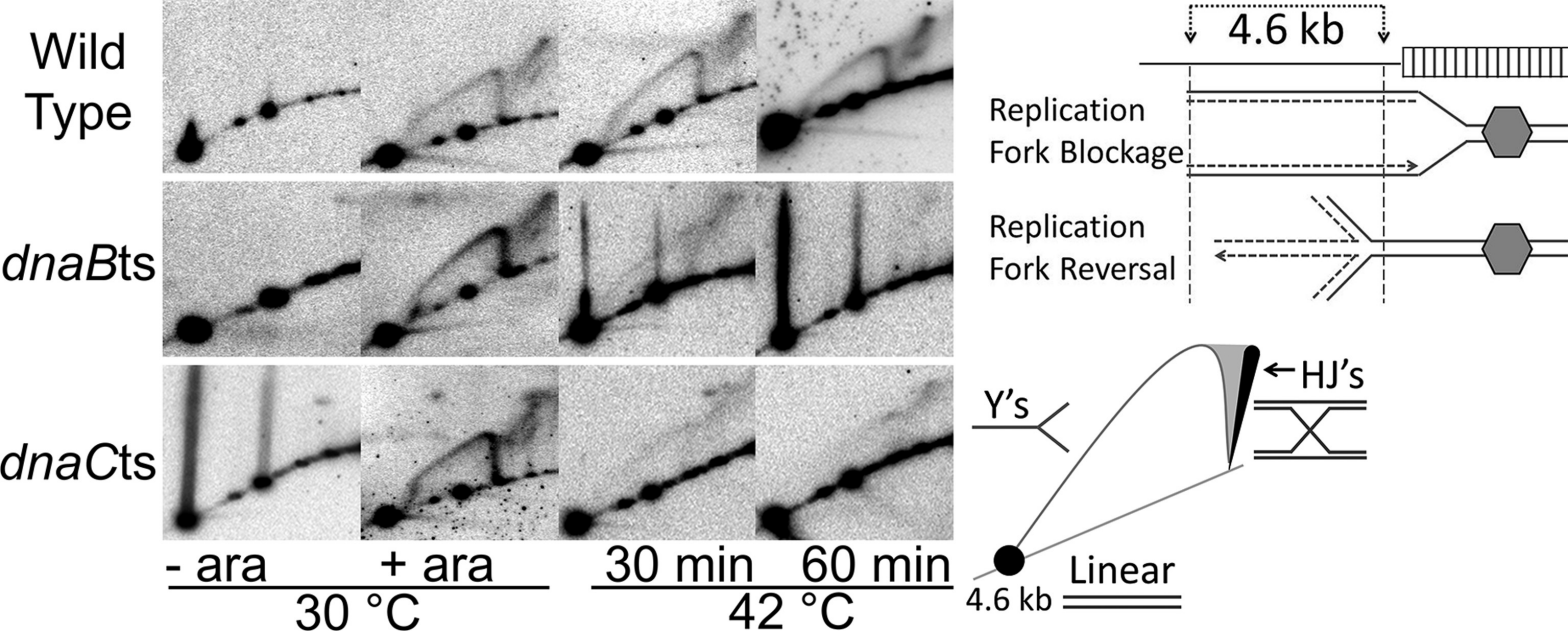
Holliday Junctions are seen upstream of the *tetO* array. 2D gel analysis of EcoRI digested DNA immediately upstream of the array following replication block (+ ara) and subsequent shift to 42°C. Schematics are of the EcoRI sites 0.9 kb and 5.5 kb upstream of the array region (indicated by arrows) and subsequent signals observed by Southern hybridisation and a radioactive probe within the 4.6 kb fragment. Holliday junction (HJ) formation is visualised as a cone signal at the top of the Y-arc and a spike from the linear DNA at the end of the Y arc.

To address whether RFR was occurring when the temperature sensitive mutants were shifted to non-permissive temperature, the structure of the DNA upstream of the array region was visualised. Duplicate samples of those analysed in Figure 3B were digested with EcoRI and the DNA subsequently analysed by 2D gel electrophoresis and Southern blot (Figure 4). This digest yields a 4.6 kb fragment, 0.9 kb upstream of the array (Figure 4). The EcoRI site closest to the array is 300 bp downstream of the EcoRV fragment, and this overlap of the fragments ensures that all DNA directly upstream of the array is visualised over the two blots. In the unblocked (- ara) samples, only linear DNA was seen for all three strains. In the blocked (+ ara) samples of all three strains, a Y-arc is visualised along with an adjacent cone signal/spike. This signal is indicative of Holliday junction (HJ) formation (29), the expected outcome of RFR; the fork has regressed towards *oriC* and the nascent DNA strands have annealed to form the four-arm HJ. The presence of the Y-arc could be due to the degradation of the 4^th^ arm of this HJ by RecBCD to reform a Y-shaped DNA structure, or may be due to replication that has restarted and the forks are proceeding through the region. The HJ signal is present at times where the replication fork block has been established (compare Figures 3B and 4), indicating the nucleoprotein block causes Holliday junction formation upstream. The Y-arc and HJ signals are also present in the wild type samples taken after 30 min and 60 min at 42°C; either HJs are formed and not processed or the signal represents a steady-state of turnover and re-formation of HJs. Faint cone signals adjacent to the Y-arc are visible at 30 min at non-permissive temperature in both the DnaBts and DnaCts mutants; a faint signal is also visible at 60 min in the DnaCts mutant. This low signal correlates to the weak blocked signal seen in Figure 3B of these samples, indicating the HJ is directly related to the formation, and processing, of the blocked signal. Therefore, the disappearance of the Y-signal (Figure 3B) reflects replication fork processing, and the HJ signal is evidence for RFR occurring. The substantial disappearance of the Y-signal in the DnaCts strain suggests that the replisome collapses in this strain within 30 min, allowing processing of the forked DNA despite all the replisome components at the fork being wild type.

Taken together the data shows that the *dnaBts* and *dnaCts* strains have their replication forks blocked by the protein–DNA Fluorescent Repressor Operator System (FROS) array and that the shift to non-permissive temperature leads to processing of the fork and may be accompanied by dissociation of some or all of the replisome. However, upon return to permissive temperature, replication is able to restart throughout the population within 10 min, and viability is not affected. Replication fork collapse, processing and restart must be occurring very efficiently in these cells. The replication fork processing by RFR appears to be a major pathway although this does not rule out that other processing is also occurring.

### The replication fork collapses at a similar rate in a wild type replisome to a temperature sensitive one

**Figure 5.**
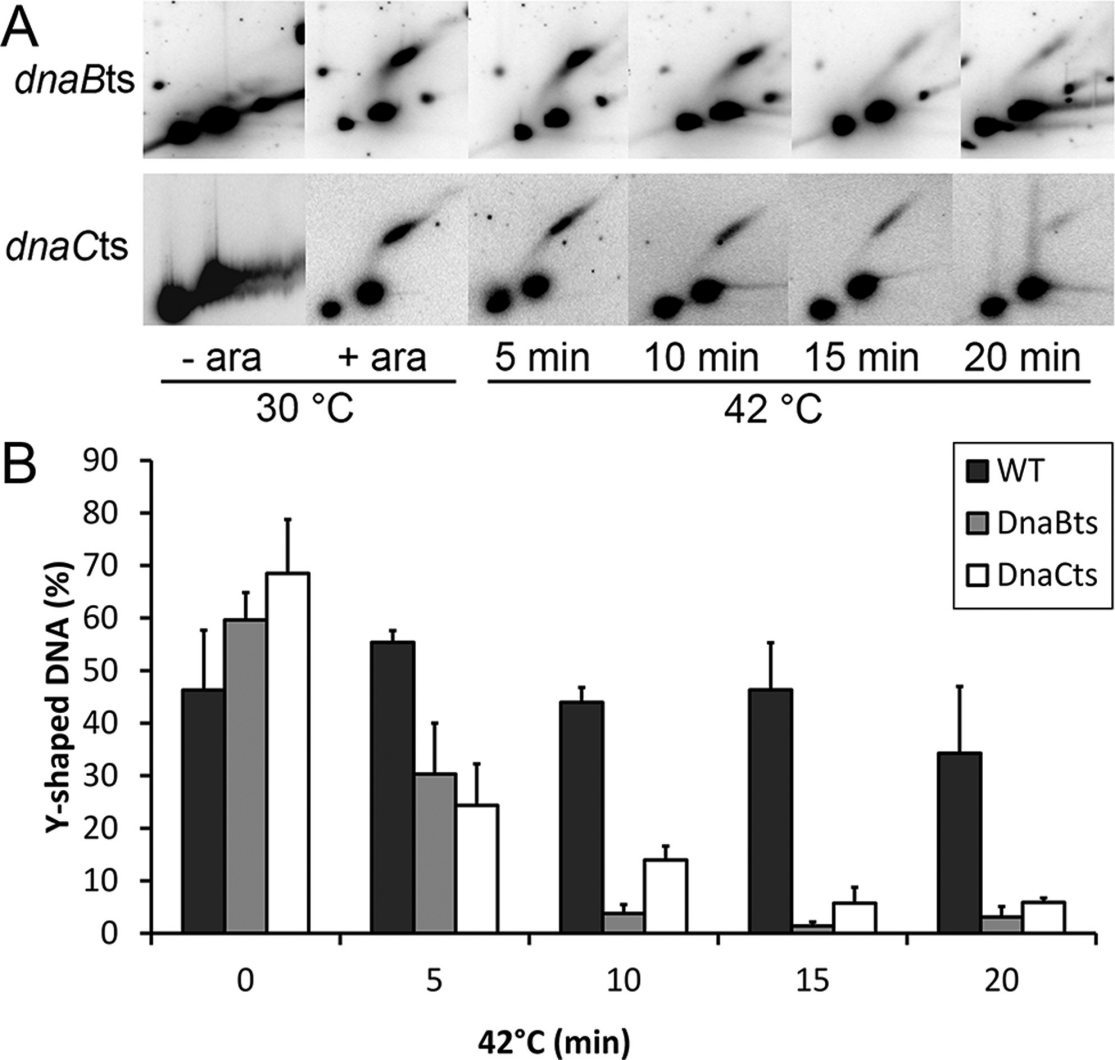
Replication fork stability at a replication roadblock. (**A**) 2D gel analysis of EcoRV digested DNA following replication block (+ ara) and subsequent shift to 42°C, a non-permissive temperature for DnaBts and DnaC(ts). (**B**) Percentage of 5.5 kb DNA within the blot located in the Y-arc.

To further determine the time it takes for a replication fork to collapse, the wild type replisome in the *dnaCts* strain was compared to one that is synthetically forced to dissociate in the *dnaBts* strain, over a shorter time frame. Cells were grown at 30°C, transferred to 42°C and samples taken at the indicated time-points for analysis by 2D gel electrophoresis (Figure 5A). Within 10 min of the shift to non-permissive temperature, only 14% of the DNA in the *dnaCts* variant remained at the blocked signal. In comparison, a wild type strain had 44% and a DnaBts strain had 4% (Figure 5B). This suggests that the *dnaBts* mutation does rapidly lead to the replication fork being processed upon inactivation by exposure to non-permissive temperature. Whilst nothing is known as to the state of the replisome in these cells, it is a reasonable assumption that the inactivation of DnaB might lead to partial or complete dissociation of the replisome from the DNA. When strains carrying the *dnaB8* allele are shifted to non-permissive temperature, DNA synthesis ceases (19,30). The A130V mutation is presumed to undergo a conformational change in response to the temperature shift leading to its dissociation from the DNA. As an integral component of the replisome, DnaB dissociation could cause at least some other components of the replisome to also dissociate. However, regardless of the occupancy of the replisome the Y-shaped DNA becomes accessible to processing proteins.

It is also clear that there is considerable collapse of the replication fork DNA in the *dnaCts* strain at the higher temperature. Furthermore, it can be seen that the decrease in the Y-shaped signal began to occur within 5 min of the temperature shift in both *dnaBts* and *dnaCts*. In a wild-type strain, the signal corresponding to blocked forks does not dissipate during the time course (Figure 5B). If the *dnaCts* mutation does not affect the stability of the stalled replisome directly, then the difference between wild type and *dnaCts* must be due to the inability of the mutant to re-load the replisome at non-permissive temperature. This indicates that a wild type replisome is not as stable as currently presumed; the wild type replisome must be continually dissociating and reassociating with the DNA at the roadblock. The ‘stable’ Y-shaped replication block signal seen previously (Figure 5B, (4)), therefore, represents the equilibrium state of replication forks that have encountered the fork and not yet collapsed, together with forks which have undergone RFR, processing and then re-loading of the replisome which then encounters the *tetO* roadblock again. This process must be in a fairly rapid equilibrium. This view is supported by the visualisation of HJs upstream in the wild type (Figure 4) showing the collapse and processing of forks is occurring.

### The half-life of a stalled replisome is less than 5 min

**Figure 6.**
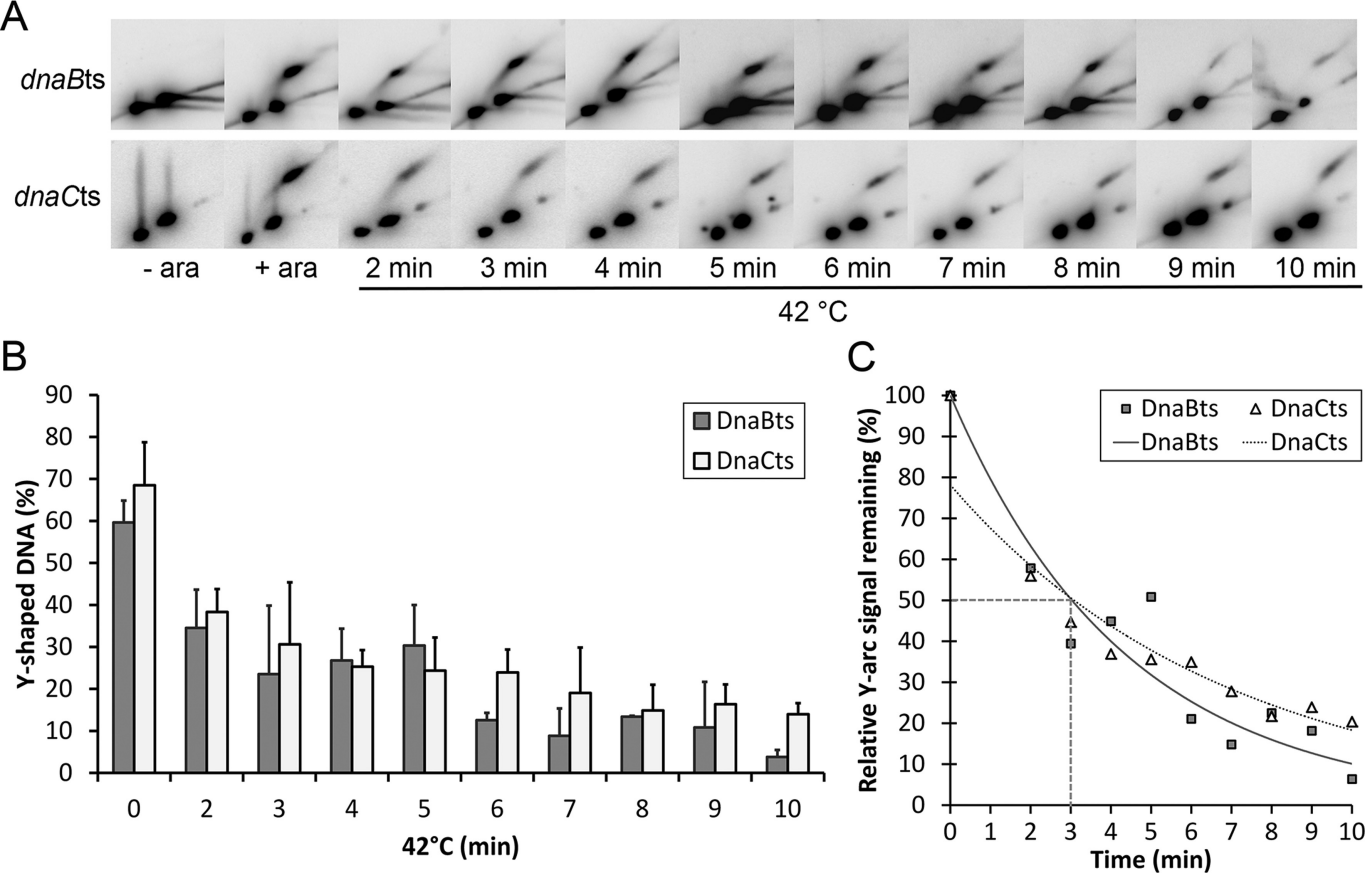
The half-life of a replisome at a nucleoprotein roadblock. (**A**) 2-D gel analysis of EcoRV digested DNA following replication block (+ ara) and subsequent shift to 42°C, a non-permissive temperature for DnaBts and DnaC(ts). (**B**) Percentage of 5.5 kb DNA within the blot located in the Y-arc. (**C**) Percentage of 5.5 kb Y-shaped DNA plotted over time with an exponential decay curve fitted.

To more precisely define the time at which the replication forks collapse, the half-life of the replisome at the roadblock was determined following a shift from 30°C to 42°C for both the *dnaBts* and *dnaCts* strains. Samples of each culture were taken at 1 min intervals and examined using 2D gel electrophoresis (Figure 6). Within 3 min, more than half of the DNA that had been present in the Y-arc of both strains was seen to revert to the size of linear DNA (Figure 6C). The calculated half-life of the replisomes *in vivo* from these experiments is 3.0 min and 3.1 min for *dnaCts* and *dnaBts* respectively. These figures are likely to be slight over-estimates of the replication fork stability because there will be a small time delay for the culture to reach non-permissive temperature upon the transfer to 42°C.

### The *dnaCts* mutation does not affect the ability of a replisome to function

A further experiment was carried out as a control to directly determine whether the *dnaCts* mutation affected the ability of a replisome to function at non-permissive temperature within this 5 min time period. Cells of all three strains had their replication blocked at the array as above. Each strain was then shifted to 42°C for 2 min, and anhydrotetracycline was added to release the replication block. The cells were kept at the non-permissive temperature for a further 10 min to allow replisomes to continue through the array if they were functional. Cells were then examined under the fluorescence microscope and the percentage that had managed to duplicate the array was determined (Supplementary Figure S3). At this time, inthe wild-type strain 81% of cells showed duplication of the array (two or more foci per cell), compared to 61% in the *dnaCts* strain, and only 17% in the *dnaBts* strain. The percentage of cells able to restart in the *dnaCts* strain agrees well with the proportion of Y-shaped DNA that was seen to remain (∼60%) after 2 min at 42°C (Figure 6). If instead, each strain was shifted back to 30°C at the point of addition of anhydrotetracycline, then they all displayed over 80% of cells with ≥2 foci after 10 min. This confirms that the inactivation of DnaCts does not prevent existing replisomes from functioning, but inactivation of DnaBts does. It also suggests that the Y-shaped DNA seen in 2D gels has a functional replisome associated with it.

## DISCUSSION

This study has determined the stability of a replication fork *in vivo* that has stalled because of a nucleoprotein block formed by an array of tetracycline repressor-operator site complexes. This FROS system is able to cause a replisome to stall in a known location on the chromosome and the replication status of the array can then be determined visually using fluorescence microscopy and verified with neutral-neutral 2D agarose gels. In wild type cells the blocked replication forks appear stable as judged by a relatively constant level of Y-shaped replication forks present at the block over time. However, when a mutant in the replicative helicase loader protein, DnaCts, is introduced the blocked signal appears stable over time at permissive temperature, but when DnaCts is inactivated at 42°C then the Y-shaped DNA rapidly disappears. In the absence of DnaC, the helicase DnaB cannot be re-loaded onto the DNA if it dissociates, and DnaB is a key protein in replisome assembly both at the replication origin and when re-loading the replisome by PriA/PriC away from the origin (31). It is not thought that DnaC itself is present at the replisome and so inactivation of the protein should not lead to changes in replisome stability or activity (Supplementary Figure S3). Therefore, the loss of the Y-shaped DNA replication fork must be due to its natural collapse over time and the failure to then re-load or re-activate the replisome without the activity of DnaC. We can estimate the half-life of the blocked replication fork to be around 3 min, which agrees well with earlier studies *in vitro* that the *E. coli* replisome has a half-life of∼5 min when it encounters a nucleoprotein block (14). It is also in agreement with the half-life observed for *in vitro* reconstituted replisomes during rolling circle replication that show a mean processivity of ∼85 kb and a speed of 535 bp/s; this means the average time an elongating replisome spends on DNA is around 2 min 40 s (32).

Using the FROS array in addition to the *dnaBts* allele, we can stop replication at a known position on the chromosome in a population of cells, and then, by temperature shift, cause the replisome to rapidly dissociate from the DNA. This could prove to be a highly useful tool for future studies on replication fork collapse and RFR, and the proteins and pathways involved in RFR and subsequent processing and reloading of the replisome.

What actually happens to the replisome when it is stalled and does it dissociate when the replication fork is processed? Previous studies using a fluorescent fusion of DnaQ have revealed that around 80% of cells show co-localisation of the replisome with the fluorescent repressor array. We have shown that 46–68% of the DNA at the array can be detected as being Y-shaped, depending upon the strain and conditions used (Figure 3B). Further, upon addition of anhydrotetracycline, the blocked array is rapidly replicated. This suggests that the majority of Y-shaped DNA signal at the block is either associated with a functional replisome that is paused and able to resume replicating once the protein roadblock is removed, or upon removal of the block, replisome reloading occurs rapidly to allow replication to resume. The re-activation of the replisome seen after 2 min at 42°C in a *dnaCts* mutant argues the majority of the Y-shaped fork is indeed associated with a stalled, but otherwise intact replisome (Supplementary Figure S3). When the replisome is inactivated using a *dnaBts* allele, the Y-shaped arc rapidly disappears and we believe that this is mostly due to RFR due to the prominent HJ signal seen upstream of the blocking array. The notion that the replication machinery has dissociated from the DNA in the strains carrying *dnaBts* or *dnaCts* alleles is supported by the observation that the addition of anhydrotetracycline to the cells after prolonged incubation at 42°C does not result in duplication of the YFP focus (Figure 1; Supplementary Figure S1). At 30°C, multiple foci are observed in these strains because anhydrotetracycline causes TetR-YFP to be released from the array enabling the fork to proceed through the former blockage and duplicate the array. In a wild type cell, this duplication is able to occur at either 30°C or 42°C. If the replisome was still present in the *dnaCts* cells at 42°C, then it should be able to continue replicating without requiring re-loading by DnaC. This is what is seen at short times of incubation at non-permissive temperature (Supplementary Figure S3), but with prolonged exposure at the higher temperature this does not occur and we infer that the replisome has left the DNA. We can be confident that the requirement for both DnaB and DnaC to be functional for re-start to occur following RFR means that the DnaB helicase must be actively re-loaded, probably via PriA. Whether the entire replisome completely dissociates or not is unknown, but it is possible that some replisome components could remain associated with the DNA whilst others (including DnaB in these experiments) dissociate. But, the complete removal of the replisome would allow unfettered access to the DNA for the subsequent repair processes and is an attractive model. Once the repair proteins have dissociated the PriA- or PriC-dependent reloading of DnaB will occur; DnaB serves as a key anchor for recruitment of the remaining replication proteins allowing the functional replisome to be reconstituted.

Using the same methodology as employed in this study it has been observed that the signal representing the stalled replication fork is stable for an extended period of time (4 h) *in vivo* (4). Given the current results and the previous absence of replisome reloading inhibition, it is almost certain that the prolonged signal that was obtained was an equilibrium view resulting from the turnover of forks; it is now known the replication forks collapse within a short timeframe (<5 min), and, therefore, the constant collapse, reloading of the replisome and reformation of the fork would not have been discernible with the previous methodology that was used (4).

The turnover of stalled replication forks is likely to also have had an effect on our results. We visually determined the replication status of the cells using fluorescence microscopy prior to inducing replication fork collapse at non-permissive temperature (Figure 1). We found 73% of cells had one focus, implying replication fork arrest, and the remaining population had two distinct foci that were well separated, suggesting replication blockage has occurred since the array was last duplicated. We would, therefore, expect to see between 73% and 100% of the DNA at the array to be Y-shaped when analysed by 2D gels. Instead, the results of the 2D gels indicated ∼50% of the 5.5 kb DNA was linear before shifting to a non-permissive temperature. This lower than expected Y-signal in the 2D gels is likely due to a combination of effects: some Y-shaped forks may have fallen apart during DNA extraction, whilst RFR would also convert some of these forks into HJs which could then migrate outside the region being probed. These would then appear as linear DNA in the array region and as HJs in the upstream region. It is also a possibility that some replication forks were able to proceed through the array but cohesion resulted in a single focus, or that some cells in the population may not have been undergoing replication at all at the time of sampling, but we believe that these would represent a minor sub-population.

Fluorescently tagged replication proteins have been shown to colocalise at positions of nucleoprotein block (4,5). A 4h persistent colocalisation of SSB at repressor induced stalled forks has been observed (4), and it is now presumed that either SSB is staying associated with the DNA or is in a steady state of association/dissociation with the DNA. The colocalisation of DnaQ (the ϵ subunit of PolIII) at a replication fork blockage (5) suggests the replisome is present at the blockage. However, 19% of those cells were not found to have DnaQ colocalised. This was reasoned to be owing to the cells being in the G1 cell cycle stage and therefore having an inactive replisome. Given the current data, we propose that at least some of that 19% of the population had undergone replisome dissociation and replication fork processing at the time of imaging. If DnaQ, and SSB, had been dissociating and reassociating, this would not have been able to be discernible with the methodology used in these studies.

From our work presented here, we conclude that the half-life of a stalled replication fork is ∼3 min. The times for the half-lives that we have obtained may vary somewhat from other studies because of the exact experimental conditions. Overproduction of TetR-YFP to obtain the roadblock occurs at 30°C and the determination of the timed collapse occurs by shifting the culture to 42°C. Activity of the proteins may, therefore, vary from what is seen in other studies where incubation of cells is often at 37°C. Nonetheless the half-life that has been obtained by us is in line with previous works that have obtained a half-life of a replication fork at a nucleoprotein blockage *in vitro* of 6 min and a half-life of 4 min of replication forks blocked by accumulation of torsional strain in the DNA (14,15). It is also in line with the calculated half-life of extending replisomes *in vitro* (32), which suggests that perhaps blockage of the replisome does not alter the rate at which the replisome falls off DNA. The authors of the earlier *in vitro* work of replication blockage from DNA bound proteins suggested that either stabilising factors were present *in vivo*, or alternatively, the replisome was being continually reloaded once it had dissociated as a way of reconciling the short half-life with the evidence at the time of a stable replisome *in vivo* (4,14). The consistency of the half-lives obtained *in vitro* and in the current study *in vivo* suggest that neither stabilising factors nor external factors that assist in the disengagement of the replisome components are present *in vivo*. This implies the rate of collapse is inherent to the stalled fork and indeed to some essential component(s) of the replisome, and that the replisome may undergo repeated rounds of re-loading to produce the apparently stable structures seen in a wild-type strain. It also implies that replication forks will very seldom manage to replicate an entire chromosome without the need for re-loading.

The current understanding of DNA replication has evolved to view the replisome as a dynamic structure with dissociation of subunits during extension (33–36). In particular, the core polymerase may dissociate and be replaced with another PolIII, or, if required, either PolII or PolIV (37). Furthermore, the lagging strand core polymerase dissociates from the DNA on completing the synthesis of an Okazaki fragment. However, although it has dissociated from the DNA, it does not necessarily dissociate from the replisome complex (27). On completion of an Okazaki fragment, the clamp loader loads a new β clamp onto the RNA primer to enable synthesis of the next fragment. Unlike the core polymerase, this clamp is highly stable with a half-life at 37°C of ∼1 h (38). Therefore, it remains possible that the short half-life of the replication fork observed here may be limited to a replisome encountering a roadblock; a blocked replisome may dissociate more readily than an actively elongating one *in vivo*. In our model of events, the replication fork stalls because the combination of the DnaB replicative helicase and the accessory helicases Rep and UvrD are unable to dissociate the upstream proteins. The replisome subsequently dissociates and RFR takes place to allow for processing and subsequent replisome reloading. The trigger for the entire replisome to dissociate is not yet known and may be innate to the DNA–replisome complex itself, nor is it known if some subunits remain associated with the DNA.

One prediction of our model is that mutants that cannot reload the replisome following replication fork collapse should show the same fork instability (rapid loss of Y-signals in 2D gels) as observed with inactivation of DnaCts. The replication forks will fall apart with the described half-life and the absence of re-loading means that replisomes would not be replaced; the equilibrium seen in wild type cells is a balance between fork collapse and re-loading. However, it has not been possible to test replication restart mutants with our current system due to their severe viability defects. *priA* and *dnaT* mutants, part of the major restart pathway in *E. coli*, are sensitive to rich media, constitutively activate the SOS response, are sensitive to UV, show poor viability and small colony size (39,40). Mutants in either *priB* or *priC* show almost no phenotype individually due to redundancy in their functions, whereas the double *priBC* mutant shows even more severe growth and viability defects than *priA* (39). Furthermore, the *priAC* double mutant is lethal. These phenotypes reflect the vital role that replication restart plays in bacteria, consistent with a replisome that has a half-life significantly shorter than the time required to completely replicate a chromosome.

The relatively low stability of a stalled *E. coli* replisome described here is in stark contrast to that of a stalled eukaryotic replisome. The previously held conclusion that the prokaryotic replisome was stable when a replication fork met a nucleoprotein blockage (4) was in part influenced by the evidence in eukaryotes where the replisome remains intact and associated with the fork at the site of the blockage (41). When the replisome stalls, Mec1/ATR is recruited to the fork by an interaction with single-stranded DNA (42). Checkpoint mediator protein complexes involving Mrc1 and Tof1 are subsequently phosphorylated and Rad53 is activated (43). The activation of the checkpoint proteins inhibits late firing of origins preventing further replication from initiating (42). Subsequent work has found that in addition to the prevention of new replication forks from being formed, individual forks that are currently replicating may also be slowed (44). Previously, the stability of the eukaryotic replisome was thought to be dependent upon checkpoint proteins that are absent in prokaryotes (41) but it has since been shown that the replisome remains intact at the fork under hydroxyurea-induced replication stress even in the absence of ATR/Rad53 proteins (44). The repair of eukaryotic DNA following replication fork stalling, including RFR, takes place seemingly with the replisome intact (45–47). A system analogous to bacterial PriA has not yet been found in eukaryotes and, therefore, if the usually stable eukaryotic replisome does dissociate from the DNA, the DnaB homolog, CMG cannot be reloaded (48). Rather, a fork from another origin of replication will replicate the DNA to completion. The cause of the difference in stability between the prokaryotic and eukaryotic systems is still unknown, but the prokaryotic replisome may just be an innately more dynamic complex than the larger eukaryotic version.

Nucleoprotein blockages such as those studied here are thought to be the major contributor to replisome stalling (3). However, other types of replication blockages, such as UV lesions, can also cause replication fork stalling or collapse. DnaB appears stably bound to DNA on encountering a lesion following UV irradiation while the polymerase subunits dissociate to allow for processing (49). However, similar to our findings with a nucleoprotein blockage, DnaC has been shown to be required for replication restart following UV irradiation, suggesting DnaB does at some point disengage from the DNA after the encounter with the lesion (24). It is uncertain what has caused the variation in these findings but it does highlight that differences in replication blockages may lead to a repair pathway distinct from our model. On encountering a UV-induced lesion, multiple repair pathways have been proposed. The replisome can bypass the lesion and reinitiate downstream, either with or without replisome reloading (50,51). Alternatively, the replisome may dissociate to allow for DNA processing, including RFR, to remove the source of the blockage (reviewed in (52)) and the extent of DNA damage may contribute to the pathway that is utilised.

This study highlights the speed with which a replication fork is processed following stalling at a replication block. These blocks are predicted to be the most common sources of impediment the replisome is likely to encounter innately (3). While further investigation is required to determine the precise extent of replisome dissociation, these results do highlight the importance and frequency of utilisation of the pathways that process these stalled forks and reload the replisome to enable the continuation of replication.

## Supplementary Material

SUPPLEMENTARY DATA
