# Supplementary material for: Stability of blocked replication forks in vivo
Source: Nucleic Acids Res. 2015 Oct 20;44(2):657–68. doi: 10.1093/nar/gkv1079 (PMC4737137; doi:10.1093/nar/gkv1079)
Supplement: SUPPLEMENTARY DATA [file supp_44_2_657__index.html]

Stability of blocked replication forks in vivo — SUPPLEMENTARY DATA 

# Stability of blocked replication forks *in vivo*

## SUPPLEMENTARY DATA

- SUPPLEMENTARY DATA
