# Supplementary material for: Stability of blocked replication forks in vivo
Source: Nucleic Acids Res. 2015 Oct 20;44(2):657–68. doi: 10.1093/nar/gkv1079 (PMC4737137; doi:10.1093/nar/gkv1079)
Supplement: SUPPLEMENTARY DATA [file supp_gkv1079_nar-01732-v-2015-File008.pdf]

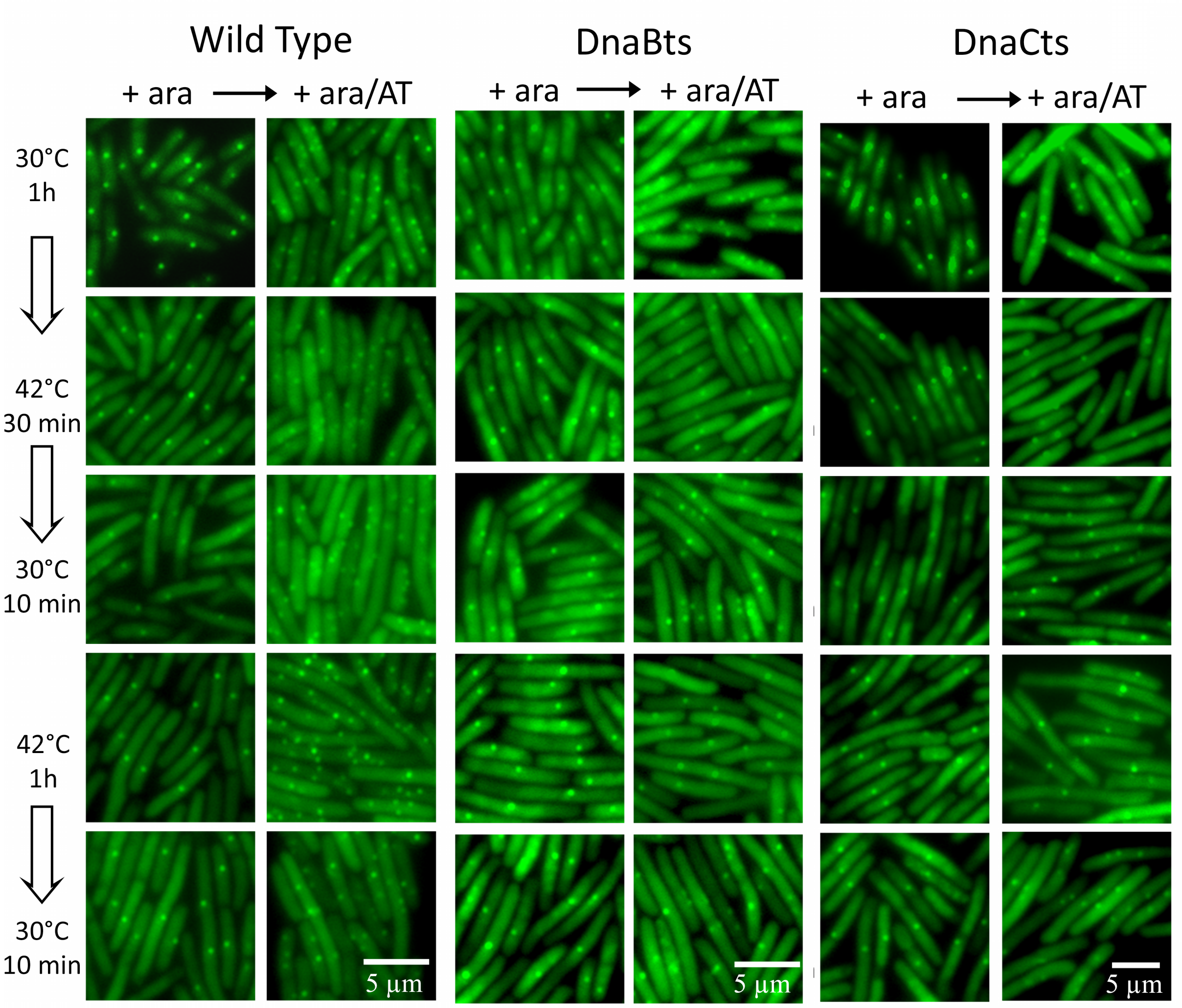

Figure S1. Representative micrographs following overproduction of TetR-YFP corresponding to samples shown in Figure 1. Wild type, DnaBts or DnaCts cells were grown at 30°C in the presence of 0.1% arabinose for 1 hour. Cells were shifted to 42°C (a non-permissive temperature for DnaBts and DnaC(ts)) for 30 minutes or 1 hour before being shifted back to permissive temperature. Anhydrotetracycline (AT) was added for 10 minutes at both temperatures and further images were taken. Multiple foci/cell are seen in cells where replication has proceeded through the array.

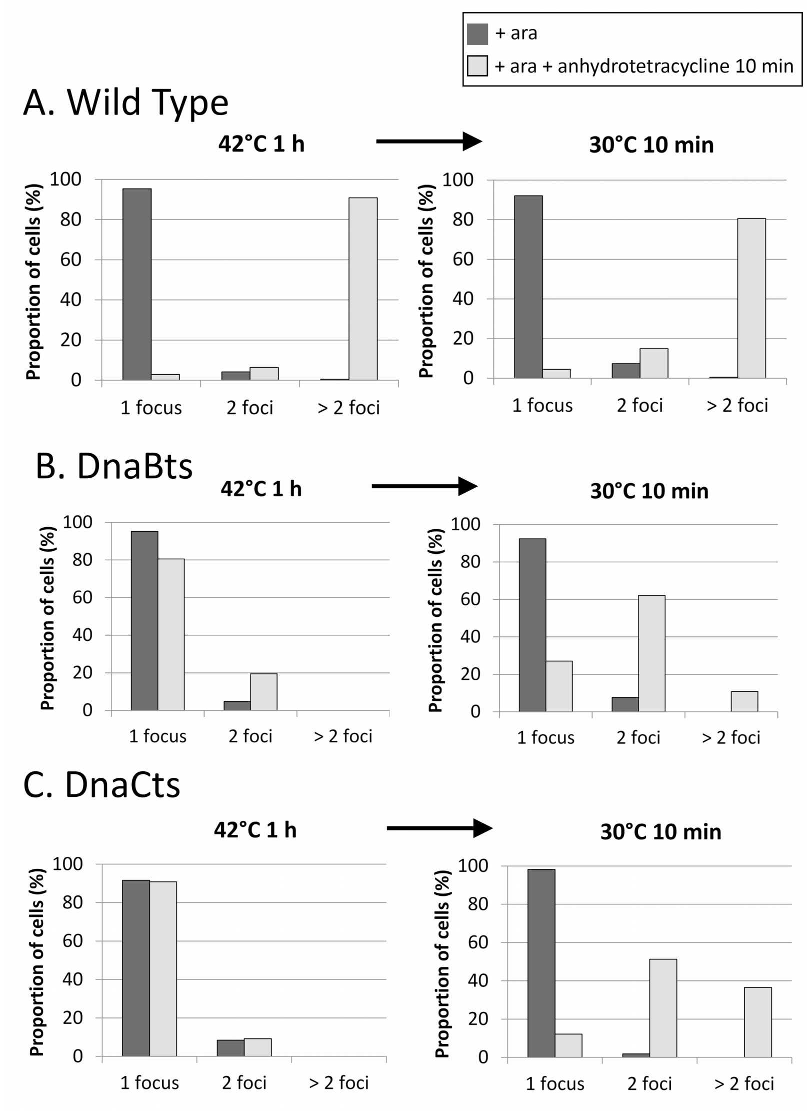

Figure S2. Proportions of cells containing single or multiple foci following overproduction of TetR-YFP. (A) Wild type, (B) DnaBts or (C) DnaCts cells were grown at 30°C in the presence of 0.1% arabinose for 1 hour (dark grey bars). Cells were shifted to 42°C (a non-permissive temperature for DnaBts and DnaC(ts)) for 1 hour before being shifted back to permissive temperature for 10 min. Anyhydrotetracycline (AT) was added for 10 minutes at both temperatures (light grey bars). This data is similar to that shown in Figure 1 except cells were kept at non-permissive temperature for a longer time (1hr compared to 30 minutes).

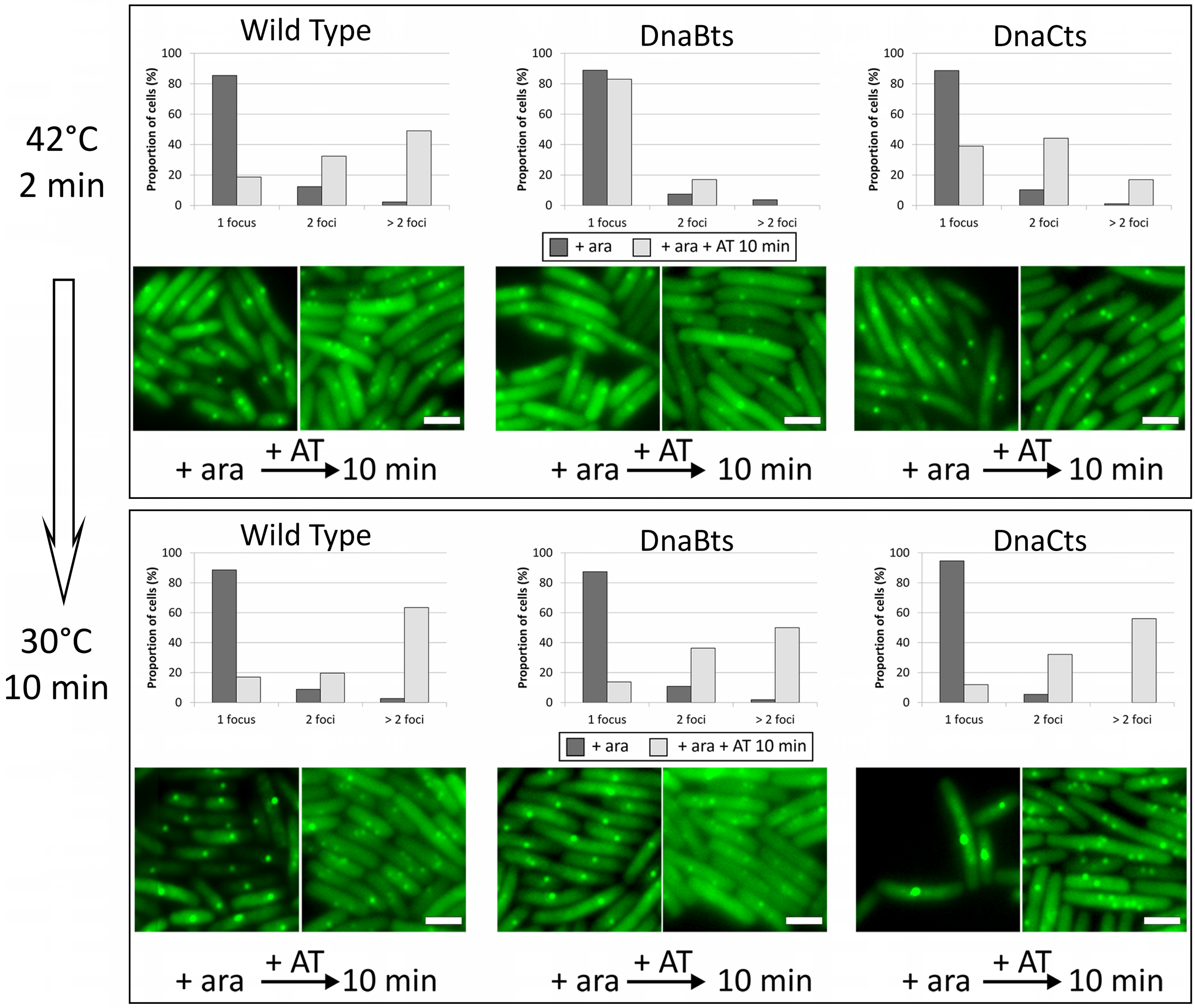

Figure S3. *dnaCts* cells are able to resume replication after release of the replication blockage at non-permissive temperature. Wild type, *dnaBts* or *dnaCts* cells were grown at 30°C in the presence of 0.1% arabinose for 1 hour and then shifted to 42°C. After 2 minutes, cells then either had anhydrotetracycline added for 10 minutes (top panel) or were shifted back to permissive temperature where a subpopulation also had anhydrotetracycline added for 10 minutes (bottom panel). The number of foci present in the cells at both temperatures was determined for cells with arabinose only (dark grey bars) or with both arabinose and anhydrotetracyline (light grey bars). Representative micrographs are shown with and without anhydrotetracycline (AT) at each temperature (scale bar = 2 μm).
